# Retention in opioid agonist treatment: a rapid review and meta-analysis comparing observational studies and randomized controlled trials

**DOI:** 10.1186/s13643-021-01764-9

**Published:** 2021-08-06

**Authors:** Jan Klimas, Michee-Ana Hamilton, Lauren Gorfinkel, Ahmed Adam, Walter Cullen, Evan Wood

**Affiliations:** 1 British Columbia Centre On Substance Use, 400-1045 Howe Street, Vancouver, BC V6Z 2A9 Canada; 2 Innovation Support Unit, Department of Family Practice, University of British Columbia, 3rd Floor David Strangway Building, 5950 University Boulevard, Vancouver, BC V6T 1Z3 Canada; 3 Department of Medicine, University of British Columbia, St. Paul’s Hospital, 608-1081 Burrard Street, Vancouver, BC V6Z 1Y6 Canada; 4 School of Medicine, University College Dublin, Health Sciences Centre, Belfield, Dublin 4, Ireland

**Keywords:** Rapid review, Opioid agonist treatment, Retention, Randomization

## Abstract

**Background:**

Although oral opioid agonist therapies (OATs), buprenorphine and methadone, are effective first-line treatments, OAT remains largely underutilized due to low retention rates and wide variation across programs. This rapid review therefore sought to summarize the retention rates reported by randomized controlled trials (RCTs) and controlled observational study designs that compared methadone to buprenorphine (or buprenorphine-naloxone).

**Methods:**

We searched four electronic databases (EMBASE, MEDLINE, Cochrane Central Register of Controlled Trials, CINAHL, up to April 2018) for RCTs and controlled observational studies that compared oral fixed-dose methadone to buprenorphine versus methadone (or buprenorphine-naloxone). Data were extracted separately for two different definitions of retention in treatment: (1) length of time retained in the study and (2) presence on the final day of a study. Separate random effects meta-analyses were performed for RCTs and controlled observational studies. Data from controlled observational studies where retention was measured as the length of time retained in the study were not amenable to meta-analysis.

**Results:**

Among 7603 studies reviewed, 10 RCTs and 3 observational studies met inclusion criteria (*n* = 5065) and compared fixed-dose oral buprenorphine with methadone. Across studies, the average retention rate was highly variable (RCTs: buprenorphine 20.0–82.5% and methadone 30.7–83.8%; observational studies: buprenorphine 20.2–78.3% and methadone 48.3–74.8%). For time period retained in the study, we observed no significant difference in treatment retention for buprenorphine versus methadone in RCTs (standardized mean difference [SMD] =  − 0.07; 95% CI − 0.35–0.21, *p* = 0.63; quality of evidence: low). For presence on the final study day, we observed no significant difference between buprenorphine and methadone treatment retention in RCTs (risk ratio [RR] = 0.89; 95% CI 0.73–1.08, *p* = 0.24; quality of evidence: low) and controlled observational studies (RR = 0.75; 95% CI 0.36–1.58, *p* = 0.45).

**Conclusion:**

Meta-analysis of existing RCTs suggests retention in oral fixed-dose opioid agonist therapy with methadone appears to be generally equal to buprenorphine (or buprenorphine-naloxone), with wide variation across studies. Similarly, a meta-analysis of three controlled observational studies indicated no difference in treatment retention although there was significant heterogeneity among the included studies. The length of follow-up did not appear to affect the retention rate.

**Systematic review registration:**

PROSPERO CRD42018104452.

**Supplementary Information:**

The online version contains supplementary material available at 10.1186/s13643-021-01764-9.

## Background

During the last decade, the ongoing opioid overdose epidemic has transitioned from being primarily heroin driven to pharmaceutical opioids, contributing significantly to drug-related mortality [1]. Recent research has shown that rates of opioid prescribing are strongly correlated with rates of opioid overdose death [2]. More specifically, in some of the most affected jurisdictions, over 70% of men and nearly 50% of women who have died of a prescription opioid overdose death did not have an active prescription in the 60 days prior to their death, suggesting the presence of significant diversion of prescription opioid medications [2].

When these analgesics first became available via unsafe prescribing practices in pain treatment, large numbers of opioid naïve persons developed prescription opioid use disorder (OUD) [3]. Illicit drug markets then capitalized on these conditions by producing unprecedented quantities of relatively cheap and illegally manufactured opioids [4]. Fentanyl contamination in the illicit drug market continues to contribute to an increase in opioid-related overdose deaths [5, 6], and a substantial proportion of OUD starts with prescription opioids [7]. In response to this crisis, evidence-based therapies for preventing overdose and treating OUD are urgently needed. Currently, the gold standard pharmacotherapies for overdose prevention are opioid agonist therapies (OATs), including buprenorphine-naloxone (Suboxone™) and methadone [8, 9]. However, only a fraction of people with OUD ever access treatment [1], and those who do are often poorly retained [10].

Although buprenorphine and methadone are effective first-line OATs [9], these effective medications remain underutilized due to low retention rates [1]. In settings where buprenorphine and methadone are widely available, many eligible persons with OUD are unable to access care, decline treatment with these medications, or—if OAT is started—are often not retained in care beyond 12 months [1, 11]. For example, recent estimates in the USA suggest that 891.8 per 100,000 people with OUD need treatment; however, only 420.3 per 100,000 people can be possibly treated with buprenorphine (and 119.9 p/100,000 with methadone) [12]. This is particularly problematic given the known increases in mortality when individuals stop OAT, due to a decline in tolerance following prolonged decreases in opioid use [13]. Clearly, there is a need to optimize attraction into and retention on first-line oral OATs to reduce opioid-related overdose and mortality. While recent reviews have demonstrated the efficacy of OAT in reducing substance-related harms, the retention rates in randomized controlled trials (RCTs) and observational studies have not been fully characterized. Due to the use of rigorous follow-up strategies, RCTs may overestimate retention on OAT therapies. This overestimation may be important for clinical care, as retention on treatment is a primary outcome of interest when prescribing buprenorphine and methadone. Estimates may be further influenced by strict inclusion criteria in RCTs, which often exclude individuals with significant comorbidities. Observational studies may therefore give a more accurate estimation of retention on OAT; however, the extent of these differences remains poorly understood.

Recently, a 2016 systematic review (55 articles) found substantial variability in OAT retention rates in randomized vs. non-randomized controlled trials (3–94% vs. 21–87%, respectively), but did not conduct a formal meta-analysis to compare retention across study designs [14]. Another Cochrane review and meta-analysis from 2014 (31 articles) evaluated buprenorphine compared to placebo and to methadone in the management of OUD for various dosing amounts and schedules (flexible vs. fixed) [9]. The review found that the effectiveness of buprenorphine was comparable to methadone but only when both were fixed, medium-to-high doses. However, there was greater effectiveness of methadone for the retention of patients for flexible and low doses. The effects of randomization on retention rates were not evaluated. This rapid review, therefore, sought to summarize the retention rates reported by RCTs and controlled observational studies that compared methadone to buprenorphine (or buprenorphine-naloxone).

## Methods

Compared to a standard systematic review, we employed the following methodological adjustments to produce this rapid review: (1) limited the search to four databases and reference lists of included articles EMBASE, MEDLINE, Cochrane Central Register of Controlled Trials, and CINAHL; (2) limited searches to the English language; and (3) limited studies to oral fixed-dose for methadone treatment. Research ethics approval was not necessary for this review. In this report, we adhered to the Preferred Reporting Items for Systematic Reviews and Meta-Analyses (PRISMA) guidelines [15] to conduct a rapid review (RR), using the evolving extension PRISMA-RR [16].

### Searching and study selection

We considered data from published RCTs and controlled observational studies that compared methadone to buprenorphine (or buprenorphine-naloxone), but excluding studies with behavioral focus and placebo comparison, until April 2018 (see Additional file 1 for the list of included and excluded studies). Only trials that defined participants as adults (≥ 18 years) with OUD were included. OUD was defined using the diagnostic criteria for OUD as defined in the Diagnostic and Statistical Manual (DSM)-IV, DSM-V, or International Statistical Classification of Diseases and Related Health Problems (ICD)-10 manuals. The considered interventions were oral fixed-dose methadone versus buprenorphine. We included controlled observational studies, randomized controlled trials (RCTs), or clinical trials (CCTs). Multiple-arm trials were included if they had at least two pharmacotherapy arms directly comparing buprenorphine and methadone.

An English language search (up to April 2018) identified studies in Cochrane Central Register of Controlled Trials, MEDLINE, CINAHL, and EMBASE. We also searched reference lists of articles considered eligible based on full report screening to identify further studies. Databases were searched using a strategy developed incorporating the filter for the identification of RCTs [22], combined with selected MeSH terms and free-text terms relating to opioid use disorder (see search strategy in Additional file 1: Table 1). We also searched reference lists of articles considered eligible based on full report screening and other relevant papers.

### Outcome measures

The primary outcome assessed was treatment retention, measured using dropout rates. This outcome was often assessed multiple times throughout the study period and measured during varied time intervals ranging from 12 to 52 weeks, depending on study length. Retention was measured as the length of time retained in treatment or study completion status, using the longest follow-up from each study (see Additional file 1 for search strategy). The level of statistical significance to assess differences between treatment and control groups was set a priori at *p* < 0.05.

### Data extraction and analysis

All citations identified by the search were independently screened based on title and abstract by two reviewers (LG, AA). Each potentially relevant study was then reviewed in full text (AA, MAH) and assessed for all inclusion criteria. Any disagreements were resolved by discussion among reviewers (LG, AA) and senior investigators (JK, EW). Relevant data from eligible articles (i.e., authors**/**country; design; participants [*N*, age, gender, diagnosis]; interventions [dosage]; and retention rates—both categorical and continuous) were then extracted (AA, LG, MAH).

### Risk of bias assessment

Study quality was assessed using the criteria and the tool from the Cochrane Handbook for Systematic Reviews of Interventions [15] by two reviewers (AA, MAH). Each trial was assessed for the risk of bias in random sequence generation and allocation concealment (i.e., selection bias). Blinding of participants and personnel (i.e., performance bias) and of outcome assessment (i.e., detection bias; objective and subjective outcomes were combined) was measured; however, since blinding was considered unlikely to affect the study outcome in this context [17], open-label studies were included. Incomplete outcome data (i.e., attrition bias) was recorded for each eligible study. Each category of bias was assigned a rating of low, high, or unclear risk using protocols from the Cochrane Handbook [15]. Observational studies were assessed for quality using an eight-item tool derived from the Joanna Briggs Institute (JBI) Cohort Study Critical Appraisal Instrument for observational studies [18]. The JBI tool considered studies on the following criteria: selection of the study groups, comparability of the groups, addressing bias and confounding factors, and ascertainment of the outcome of interest.

### Data analysis

For the meta-analysis, dichotomous outcome measures (treatment retention defined as present on the last day of treatment) were analyzed by calculating the risk ratio (RR) for each trial, with uncertainty in each result expressed via 95% confidence intervals (CIs). Continuous outcomes, such as the number of days retained in treatment, were analyzed by calculating the mean difference (MD) between experimental and control groups. In addition, the effect of follow-up period length on retention was characterized by grouping studies using the length of follow-up and then comparing study retention rates. Review Manager (v.5.3) was used to conduct the meta-analyses.

Information on missing data was collected where possible from study authors. If study authors were unable to supply this information, missing data were obtained or calculated from values in the primary studies according to suggested procedures in the Cochrane Handbook for Systematic Reviews of Interventions [15].

Given the expected heterogeneity of results among studies due to differences in population and intervention type, we employed a random effects meta-analytic model. The *I*-squared (*I*^2^) statistic was employed to test the presence of heterogeneity between trials.

### Protocol and registration

The review has been registered with the PROSPERO register of systematic review protocols on August 8, 2018 (registration No. CRD42018104452, web address: https://www.crd.york.ac.uk/prospero/display_record.php?RecordID=104452). Since the registration, the following deviations from the protocol have been applied to accommodate reduced staff availability: the intervention under study was re-defined as fixed-dose buprenorphine or methadone, “no intervention” has been removed from the definition of the comparator, and the review has been completed as a rapid review without a meta-regression.

## Results

**Fig. 1:**
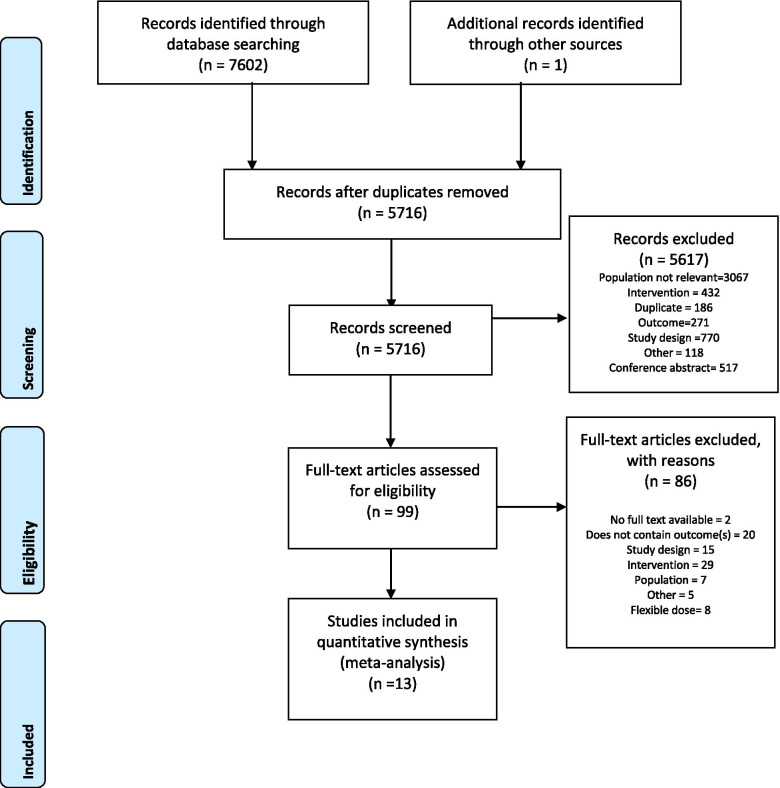
Flowchart of studies. Comparison of retention in oral fixed-dose methadone versus buprenorphine Preferred Reporting Items for Systematic Reviews and Meta-Analyses (PRISMA) flow diagram. Creative Commons Attribution License 4.0, adapted with permission [39]

A total of 7603 records were identified as potentially eligible, with 5716 records remaining after de-duplication. After title and abstract screening, 99 full texts were assessed for eligibility. Eight studies were excluded from the present analysis, as they utilized flexible doses. A total of 13 full texts (*N* = 5065) met the inclusion criteria (Fig. 1), including 10 RCTs [19–28] and three observational studies [29–31]. Among the 10 included RCTs (*n* = 1465), the mean doses of methadone and buprenorphine used were 60.46 mg/day and 7.79 mg/day, respectively. Suboxone was used in 2 RCTs with a mean dose of 8.50 mg/day. The formulations of buprenorphine that were used included sublingual buprenorphine tablets [19, 20, 22, 25, 27, 28] and buprenorphine-naloxone [21, 24]. The treatment was on average 24.4 weeks long and the retention rate varied widely (buprenorphine range 20.0–82.5% and methadone range 30.7–83.8%).

Among the three included controlled observational studies (*n* = 3600), the mean doses of methadone and buprenorphine used were 69.27 mg/day and 8.84 mg/day, respectively. The formulations of buprenorphine that were used included sublingual buprenorphine (including Subutex) [31]. The treatment was on average 29.3 weeks long and the retention rate varied widely (buprenorphine range 20.2–78.3% and methadone range 48.3–74.8%).

Quality assessments for each study are presented in Table 1. All RCTs were found to be low to unclear risk of bias for incomplete outcome data. There was an unclear or high risk of bias relating to blinding of outcome assessments, allocation concealment, and random sequence generation which was particularly noteworthy in the studies by Eder et al. and Ahmadi et al. [20, 25]. For controlled observational studies, two studies [29, 31] were rated at moderate risk and one at low risk of bias [30].

**Table 1:** Characteristics of included studies

Study/Country	Design	Participants	Interventions	Outcomes	Risk Rating
Ahmadi 2003A Iran	Randomized controlled trial Double blind Duration: 18 weeks	N = 164 Mean age: 31.4 years old Buprenorphine, 33.7 years old Methadone Male: 100% Opioid Dependence (DSM-IV criteria)	(1) Buprenorphine 1 mg/day (2) Buprenorphine 3 mg/day (3) Buprenorphine 8 mg/day (4) Methadone 30 mg/day	Retention as measured by completion rates	
Ahmadi 2003B Iran	Randomized controlled trial Open label Duration: 24 weeks	N = 204 Mean age: 31.2 years old Male: 100% Opioid Dependence (DSM-IV criteria)	(1) Methadone 50 mg/day (2) Buprenorphine 5 mg/day	Retention as measured by completion rates	
Eder, 1998 Austria	Randomized controlled trial Open-label Duration: 24 weeks	N = 34 Mean age: 26.35 years old Sex not specified Opioid dependence (criteria not specified)	(1) Buprenorphine 7.3 ± 1.8 mg (2) Methadone 65.6 mg ± 26.6 mg	Retention as measured by dropout rates/completion of study phase Abstinence from opioids Toxicology Urine Samples	
Gerra, 2004 Italy	Observational, non-randomized study Duration: 12 weeks	N = 154 Mean age: 32.5 years old Male: 74.7% Heroin dependence (criteria not specified)	(1) Methadone 81.5 ± 35.4 mg (2) Buprenorphine 9.2 ± 3.4 mg	Retention rate as measured using a survival analysis Abstinence from illicit drugs Psychiatric comorbidity	Moderate Risk
Johnson, 1992 USA	Randomized controlled trial Double blind Duration: 17 weeks	N = 162 Mean age: 33.4 years old Buprenorphine-8, 32.7 years old Methadone-20, 33.1 years old Methadone-60 Males: 69.8% Opioid addiction	(1) Buprenorphine 8 mg/d (2) Methadone 20 mg/d (3) Methadone 60 mg/d	Retention in treatment as measured by percent of patients retained in each treatment Urine samples negative for opioids Failure to maintain abstinence	
Kamien, 2008 USA	Randomized controlled trial Double dummy Duration: 17 weeks	N = 268 Mean age: 37.2 years old Buprenorphine-Naloxone-8, 38.9 years old Buprenorphine-Naloxone-16, 40.3 years old Methadone-45 38.1 years old Methadone-90 Male = 70.7% Buprenorphine-Naloxone-8, 70.7% Buprenorphine-Naloxone-16, 80.8% Methadone-45, 65.8% Methadone-90 Opioid dependence (DSM-IV criteria)	(1) Buprenorphine-naloxone 8–2 mg (2) Buprenorphine-naloxone 16–4 mg (3) Methadone 45 mg (4) Methadone 90 mg	Retention in treatment as measured by weeks in treatment Opioid abstinence Medication compliance Non-opioid drug use	
Kosten, 1993 USA	Randomized clinical trial Double blind Duration: 24 weeks	N = 140 Mean age: 32 years old Methadone-35, 32 years old Methadone-65, 33 years old Buprenorphine-2, 32 years old Buprenorphine-6, Male = 73% Opioid dependence (DSM-III-R criteria)	(1) Methadone 35 mg (2) Methadone 65 mg (3) Buprenorphine 2 mg (4) Buprenorphine 6 mg	Treatment retention as measured by weeks in treatment Urine toxicology Self-reported use Withdrawal symptoms Addiction Severity Index	
Ling,1996 USA	Randomized controlled trial Double blind Duration: 52 weeks	N = 225 Mean age: 41.5 years old Buprenorphine-8 40.8 years old Methadone-30 40.1 years old Methadone-80 Male = 79.6% Opioid dependence (DSM-III-R criteria)	(1) Buprenorphine 8 mg (2) Methadone 30 mg (3) Methadone 80 mg	Retention as measured by weeks in treatment, and as measured by percent of patients retained in each treatment Opioid use Opioid craving Adverse events	
Maremmani, 2007 Italy	Observational study Duration: 52 weeks	N = 213 Mean age: 31 years old Opioid dependence (DSM-IV criteria) Male = 82.6%	(1) Buprenorphine 5.1 mg (2) Methadone 61.68 mg	Retention in treatment as mesured by percent of patients retained in each treatment Longitudinal analysis Adverse events	Low Risk
Otiashvili, 2013 Georgia	Randomized Controlled Trial Duration = 12 weeks	N = 80 Mean age: 33.7 years old Opioid dependence (ICD-10 criteria)	(1) Methadone 39 ± 17.8 mg (2) Buprenorphine (Suboxone) 8.5 ± 3.5 mg	Retention in treatment as measured by weeks in treatment, and number of patients retained in each treatment Urine toxicology HIV risk injection behaviours Adverse events	
Pani, 2000 Italy	Randomized Controlled Trial Double blind Duration = 24 weeks	N = 72 Mean age: 28 years old Male: 86.1% Heroin addiction of at least 2 years (DSM-IV criteria for opioid dependence)	(1) Buprenorphine 8 mg (2) Methadone 60 mg	Retention in treatment as measured by proportion of patients retained in each treatment Urine toxicology Predictors of treatment compliance Adverse events	
Proctor, 2014 USA	Retrospective chart review Duration: 24 weeks	N = 3233 Mean age: Male: 55.9% Methadone, 57.8% Buprenorphine (Suboxone), 50.05% Buprenorphine (Subutex)	(1) Methadone 64.64 mg/d (2) Buprenorphine (Suboxone) 9.75 mg/d (3) Buprenorphine (Subutex) 12.21 mg/d	Retention in treatment as measured by length of stay in days, and percent of patients retained in each treatment UDS Findings	Moderate Risk
Schottenfeld, 1997 USA	Randomized controlled trial Double blind Duration: 24 weeks	N = 116 Mean age: 32.6 years old Methadone-65, 32.6 years old Buprenorphine-12, 31.6 Methadone-20, 33.7 Buprenorphine-4 Male: 57% Methadone-65, 69% Buprenorphine-12, 72% Methadone-20, 77% Buprenorphine-4	(1) Methadone 20 mg (2) Methadone 65 mg (3) Buprenorphine 4 mg (4) Buprenorphine 12 mg	Treatment retention as measured by completion rates Urine toxicology	

Risk rating legend: A: random sequence generation (selection bias); B: allocation concealment (selection bias); C: blinding of participants and personnel (performance bias); D: blinding of outcome assessment (detection bias); E: incomplete outcome data (attrition bias); amber circle: unclear

### Meta-analysis results

**Fig. 2:**
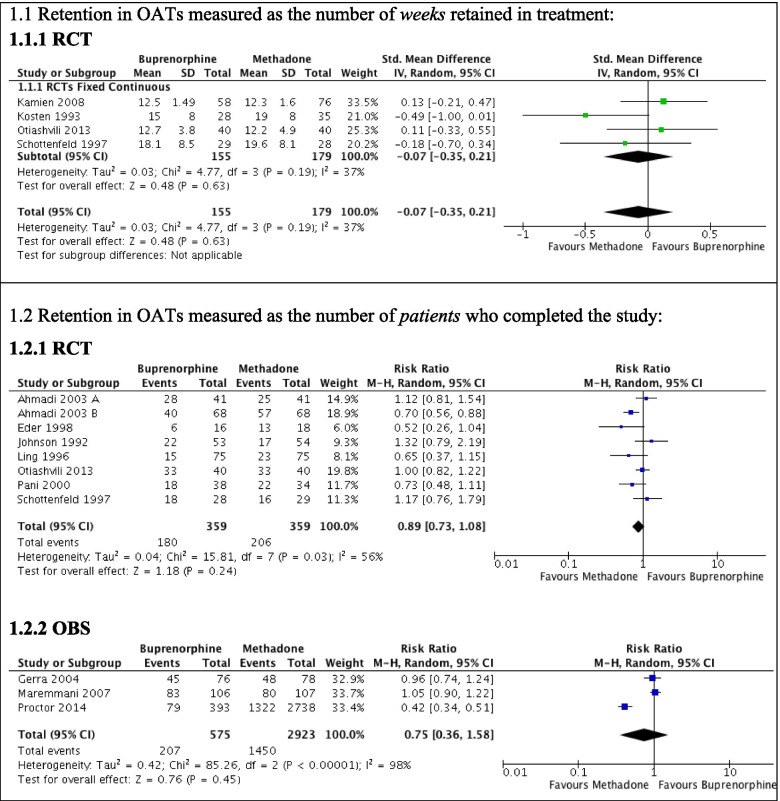
; CI confidence intervalForest plot of retention in fixed-dose oral opioid agonist treatment (OAT) assessed with observational study designs (OBS) compared with retention assessed in randomized trials (RCT)

Data are presented separately for two different definitions of retention in treatment: (1) length of time retained in the study and (2) presence on the final day. When retention was defined as the length of time (weeks) retained in the study (Fig. 2 (1.1.1)), there was no difference in the effects of buprenorphine and methadone on treatment retention evaluated in RCTs (− 0.07, 95% confidence interval [CI] − 0.35–0.21, 4 studies, *n* = 334, *I*-squared [*I*^2^] = 37%). This pattern was consistent when retention was defined as presence on final day in both RCTs (0.89 95% CI 0.73–1.08, 8 studies, *n* = 718, *I*^2^ = 56%, Fig. 2 (1.2.1)) and controlled observational studies (0.75, 95% CI 0.36–1.58, 3 studies, *n* = 3498, *I*^2^ = 98%, Fig. 2 (1.2.2)). The data for non-randomized controlled studies, where retention was measured as the length of time (weeks) retained in the study, were not amenable to meta-analysis. Most studies were rated at an overall moderate to high risk of bias, there was a substantial heterogeneity between studies, and the overall quality of the included evidence was rated as low, which is an important limitation to the generalizability and robustness of the results.

### Sub-analysis results

**Fig. 3:**
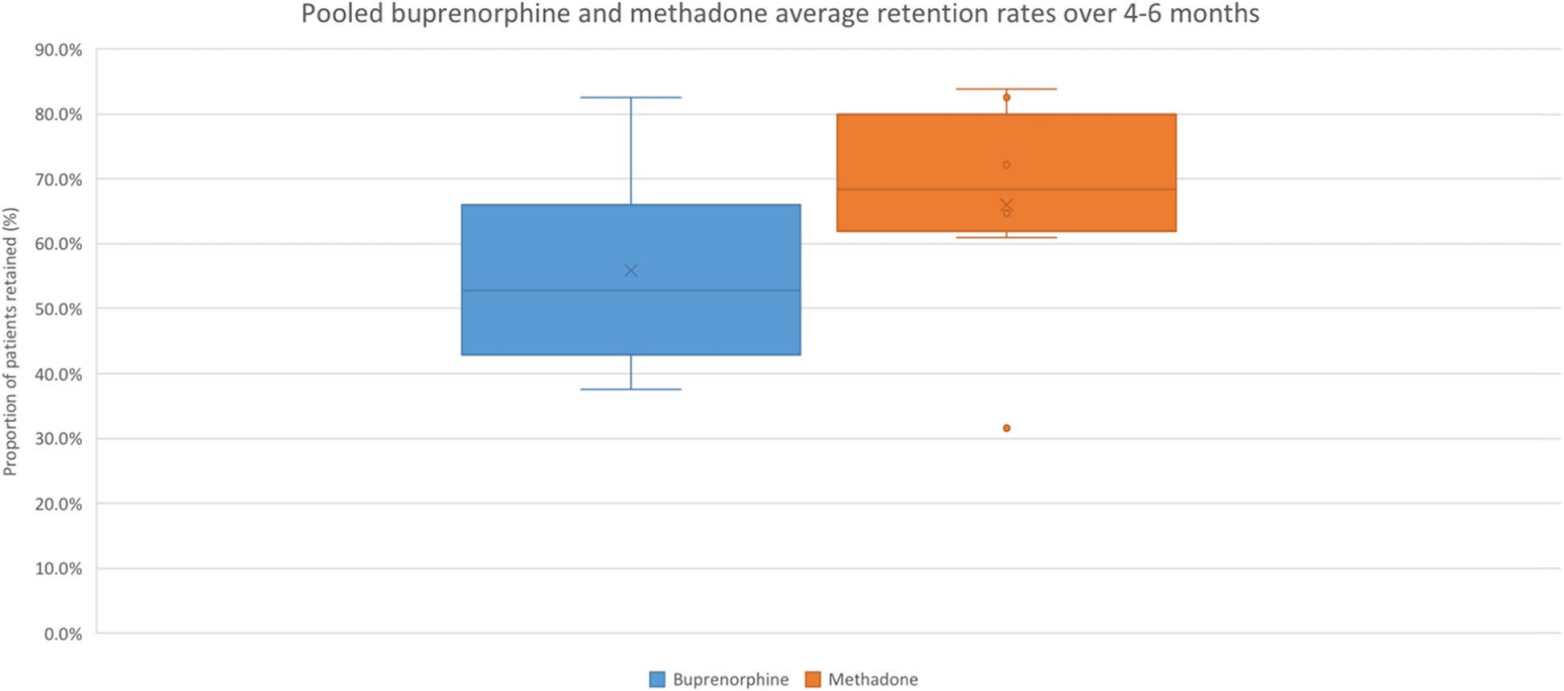
Boxplots of mean retention rate for randomized controlled trials (RCTs), 4–6 months of follow-up. Retention rates are for buprenorphine and methadone, where the retention rate was measured as the number of patients who completed the study. All studies had follow-up rates between 4 and 6 months

The mean retention rates for buprenorphine and methadone are shown in Fig. 3. A sub-analysis was completed to compare the effect of the follow-up period duration on the retention rate measured as a categorical variable (number of patients who completed the study) for both buprenorphine and methadone. Studies were grouped in follow-up period ranges (1–3 months, 4–6 months, 7–9 months, and 10–12 months). Boxplots were completed for groupings with more than one study. Therefore, only studies with follow-up lengths between 4 and 6 months were plotted [19–21, 23–25, 27, 28, 32]. The mean weighted retention rate was determined separately for randomized controlled trials using buprenorphine and methadone. The mean weighted retention rate and 95% confidence interval (CI) for studies with follow-up periods between 4 and 6 months using buprenorphine was 57.3% (95% CI = 53.5%, 61.1%) and using methadone was 65.5% (95% CI = 60.5%, 70.5%). Analyzing the retention rates once studies were grouped by follow-up length had no effect on the retention rates.

## Discussion

The findings of this rapid review and meta-analyses suggest similar retention rates on oral fixed-dose methadone and buprenorphine (or buprenorphine-naloxone), with little difference between RCTs and controlled observational studies published up to April 2018. Additionally, our findings indicate that the length of follow-up does not affect the retention rate.

Our findings align with and further corroborate the conclusions of previous systematic reviews of oral OATs for OUD in three specific ways [2, 9, 14]. First, all of these reviews found variable rates of average treatment retention across the included studies with little difference between buprenorphine and methadone [33] and little evidence of better retention rates in naturalistic studies with cohort design [14]. Second, except for the RCT-focused review by Mattick et al. [9], the reviews did not pool the data from RCTs and observational studies in separate meta-analyses and did not review literature post-2014 (e.g., Timko et al. reviewed studies between 2010 and 2014) [14]. Third, although we excluded placebo-controlled RCTs, which were included in Mattick et al.’s Cochrane review [9], we did not further restrict our review to specific populations, or countries, such as the review by Timko et al. that studied low- and middle-income countries [14]. In sum, we believe that our review provides important data on retaining participants in fixed-dose oral OAT and on differentiating retention rates in controlled studies with or without randomization of participants into treatment groups.

From a program evaluation perspective, a key contribution of this review to the wider literature is the very little difference in OAT treatment retention between randomized controlled trials (RCTs) and non-randomized but controlled observational studies. Similar findings have been reported in a 2014 Cochrane overview of 15 methodological reviews (1583 meta-analyses that covered 228 different medical conditions) assessing the impact of study design (including RCTs versus observational study designs) on the effect measures estimated [34]. While the authors, Anglemyer and colleagues, did not include reviews of substance-use interventions and excluded reviews of observational studies that had used some form of concurrent allocation, they found no effect estimate differences between observational studies and RCTs. In agreement with Anglemyer et al., we too conclude that factors other than randomization should be considered when examining differences between RCTs and observational studies in the substance-use research literature. Although this literature is still evolving to allow the drawing of firm conclusions regarding interventions for increasing retention in oral OAT [35], a rapid evaluation and scale up of novel effective OATs as part of overdose emergency response must now become a priority. OAT remains the key route for reducing overdose mortality, but non-use (due to either inability to access or non-interest in existing OAT models), or discontinuation, is the key issue that contributes to mortality for persons with OUD [13].

The rapid review has several limitations. First, only four databases were searched, and the limitation to only studies of English language published up to April 2018 provided a further restriction on the search results. Second, because of the nature of rapid reviews [36], some studies were missed due to the employed inclusion criteria. As such, the present rapid review was aligned with the evolving standards of rapid reviews [36, 37], which are not as rigorous as systematic reviews. Third, our analysis should be interpreted with caution due to its a priori narrow focus on fixed-dose oral methadone and buprenorphine. A fixed dose is probably the least used scenario in the real world [9] whereas trials use fixed doses [38]. The data for fixed-dose observational studies, where retention was measured as a continuous variable, were not amenable to meta-analysis, as this measure was not reported in more than three studies. Fourth, the longest follow-up periods in each study were used for data analysis; however, the length of these follow-up periods varied widely. Fifth, the included studies also utilized a variety of study designs, different doses, various formulations of buprenorphine, varying measures of retention, and varying numbers of treatment arms. Here, only treatment arms that were relevant to the desired comparison were analyzed. Finally, while oral OAT is effective for many patients, the observed low overall retention rates suggest further examination of methods to optimize OAT retention is necessary.

## Supplementary Information

**Additional file 1: i. eTable 1**. Search Strategies. **ii. eTable 2**. Inclusion/Exclusion Criteria. iii. eReferences – Included and excluded studies.

## Data Availability

The datasets used and/or analyzed during the current study are available from the corresponding author on reasonable request.

## Abbreviations

OUD: Opioid use disorder
OAT: Opioid agonist treatment
RCT: Randomized controlled trial
OBS: Observational study
DSM: Diagnostic and Statistical Manual
ICD: International Statistical Classification of Diseases and Related Health Problems
PRISMA: Preferred Reporting Items for Systematic Reviews and Meta-Analyses
CCTs: Clinical trials
RR: Risk ratio
CI: Confidence interval
MD: Mean difference
