# Supplementary material for: Retention in opioid agonist treatment: a rapid review and meta-analysis comparing observational studies and randomized controlled trials
Source: Syst Rev. 2021 Aug 6;10:216. doi: 10.1186/s13643-021-01764-9 (PMC8348786; doi:10.1186/s13643-021-01764-9)
Supplement: Supplementary file 1 — Additional file 1: i. eTable 1. Search Strategies. ii. eTable 2. Inclusion/Exclusion Criteria. iii. eReferences – Included and excluded studies. [file 13643_2021_1764_MOESM1_ESM.docx]

## **Supplementary Online Content**

## Klimas, J., Hamilton, M.A., Gorfinkel, L.G., Adam, A., Cullen, W., Wood, E. Retention in opioid agonist treatment: A rapid review and meta-analysis comparing observational studies and randomized controlled trials. *BMC Systematic Reviews*

## i. eTable 1. Search Strategies

## ii. eTable 2. Inclusion/Exclusion Criteria

## iii. eReferences – Included and excluded studies

## This supplementary material will be provided by the authors to give readers additional information about their work.

**i. Search Strategy**

**eTable 1: Opioid Agonist Therapy searches April 2018**

**Medline**

| 1 | exp opiate addiction/ | 22862 | **Population** |
| --- | --- | --- | --- |
| 2 | (opioid$ or opiat$ or drug or substance or abuse$ or addict$ or depend$ or disorder$).tw. | 3952914 |  |
| 3 | exp drug dependence/ | 258757 |  |
| 4 | exp opiate substitution treatment/ | 2189 |  |
| 5 | or/1-4 | 4065391 |  |
| 6 | exp Patient Compliance/ | 68506 | **Outcomes** |
| 7 | Patient Dropouts.tw. | 38 |  |
| 8 | Retention.tw. | 154769 |  |
| 9 | exp "length of stay"/ | 76570 |  |
| 10 | exp patient care/ | 861218 |  |
| 11 | (drop-out$ or dropout$ or retention or adherence or alliance or attrition$).tw. | 281124 |  |
| 12 | (completion adj2 (rate$ or treatment$)).tw. | 9307 |  |
| 13 | (patient$ adj2 return$).tw. | 11733 |  |
| 14 | ((patient$ or client$ or treatment$) adj2 complian$).tw. | 18277 |  |
| 15 | (length$ adj2 stay).tw. | 47501 |  |
| 16 | (continu$ adj3 care).tw. | 18276 |  |
| 17 | or/6-16 | 1223401 |  |
| 18 | Randomized Controlled Trials as Topic/ | 116841 |  |
| 19 | randomized controlled trial/ | 463009 | **Study design** |
| 20 | Random Allocation/ | 94652 |  |
| 21 | Double Blind Method/ | 146340 |  |
| 22 | Single Blind Method/ | 25307 |  |
| 23 | clinical trial/ | 510754 |  |
| 24 | exp Clinical Trials as topic/ | 314947 |  |
| 25 | clinical trial, phase i.pt. | 18214 |  |
| 26 | clinical trial, phase ii.pt. | 29350 |  |
| 27 | clinical trial, phase iii.pt. | 13951 |  |
| 28 | clinical trial, phase iv.pt. | 1554 |  |
| 29 | controlled clinical trial.pt. | 92463 |  |
| 30 | multicenter study.pt. | 235057 |  |
| 31 | randomized controlled trial.pt. | 463009 |  |
| 32 | clinical trial.pt. | 510754 |  |
| 33 | or/18-32 | 1241435 |  |
| 34 | (clinical adj trial$).tw. | 309232 |  |
| 35 | ((singl$ or doubl$ or treb$ or tripl$) adj (blind$3 or mask$3)).tw. | 157203 |  |
| 36 | PLACEBOS/ | 33973 |  |
| 37 | placebo$.tw. | 195952 |  |
| 38 | randomly allocated.tw. | 24356 |  |
| 39 | (allocated adj2 random$).tw. | 27398 |  |
| 40 | or/34-39 | 556126 |  |
| 41 | 33 or 40 | 1464576 |  |
| 42 | Epidemiologic studies/ | 7712 |  |
| 43 | exp case control studies/ | 921302 |  |
| 44 | exp cohort studies/ | 1750857 |  |
| 45 | Case control.tw. | 108423 |  |
| 46 | Cohort analy$.tw. | 6256 |  |
| 47 | Longitudinal.tw. | 205845 |  |
| 48 | Retrospective.tw. | 428819 |  |
| 49 | Cross sectional.tw. | 279774 |  |
| 50 | Cross-sectional studies/ | 268006 |  |
| 51 | or/42-50 | 2505342 |  |
| 52 | 41 or 51 | 3616016 |  |
| 53 | Case Study/ | 1881245 |  |
| 54 | case report.tw. | 271639 |  |
| 55 | abstract report/ or letter/ | 988797 |  |
| 56 | Conference abstract.tw. | 195 |  |
| 57 | Editorial.tw. | 41164 |  |
| 58 | Letter.tw. | 71070 |  |
| 59 | Note.tw. | 64330 |  |
| 60 | or/53-59 | 2896716 |  |
| 61 | Morphine/ | 36808 | **Intervention** |
| 62 | Methadone/ | 11604 |  |
| 63 | exp Buprenorphine/ | 4633 |  |
| 64 | Heroin/ | 5318 |  |
| 65 | hydromorphone/ | 1173 |  |
| 66 | (heroin or methadone or narcot$ or buprenorphine or oxycodone or oxycontin or hydrocodone or hydromorphone or codeine or fentanyl or meperidine or oxymorphone or propoxyphene or tramadol).tw. | 68047 |  |
| 67 | or/61-66 | 102741 |  |
| 68 | 5 and 17 and 52 and 67 | 3563 |  |
| 69 | 68 not 60 | 3506 |  |
| 70 | limit 69 to (english language and humans) | 3153 | **Final** |

**The Cochrane Central Register of Controlled Trials**

| 1 | Opioid-Related Disorders/ | 898 | **Population** |
| --- | --- | --- | --- |
| 2 | (drop-out$ or dropout$ or retention or adherence).tw. | 28355 |  |
| 3 | Patient Dropout/ | 1764 |  |
| 4 | exp Patient Compliance/ | 10685 |  |
| 5 | 2 or 3 or 4 | 36441 |  |
| 6 | Opiate Substitution Treatment/ | 244 |  |
| 7 | Morphine/ | 3864 | **Intervention** |
| 8 | Methadone/ | 950 |  |
| 9 | Buprenorphine/ | 726 |  |
| 10 | Heroin/ | 271 |  |
| 11 | hydromorphone/ | 206 |  |
| 12 | 7 or 8 or 9 or 10 or 11 | 5517 |  |
| 13 | 1 or 6 | 986 |  |
|  | 5 and 12 and 13 | 223 | **Final** |

**ii. eTable 2:** **Inclusion/Exclusion Criteria**

| **Inclusion Criteria** | **Exclusion Criteria** |
| --- | --- |
| **Population**   - Opioid agonist therapy (OAT) naïve patients enrolled in treatment for opioid use disorder (OUD) - Patients must have a defined opioid addiction or abuse. | **Population**   - Patients that are not diagnosed with OUD or not enrolled in a OUD treatment program - OAT experienced patients - Pregnant Women |
| **Intervention**   - Any intervention, that is specifically and exclusively for the treatment of OUD, including the following:   - Methadone   - Buprenorphine/naloxone     - Slow-release oral morphine   - Any other pharmaceutical maintenance therapy | **Intervention**   - Interventions that are not pharmaceutical maintenance therapies OUD - Single arm with no comparison - Detoxification studies - Studies that compare treatment centres |
| **Comparator**   - Any type of the interventions, such as the interventions listed above | **Comparator**   - Interventions that are not OAT and aimed exclusively at the treatment of OUD |
| **Outcomes**  **Primary outcome**   - Studies reporting retention for both intervention and control groups. Retained or not retained for the full length of treatment. For the purposes of this review, “retention” is defined as”   - Retention as a continuous value such as the number of days a patient continued in treatment until the last day of receiving an intervention receipt (also reported as weeks).   - As a binary outcome such as the percentage of patients who completed their treatment course   - As the reported number of patients who received the treatment for a predefined number of treatment days. | **Outcomes**   - Studies not assessing retention or adherence and compliance |
| **Study Design**   - Studies that report any measurement on rates of drop out or retention in any OUD treatment will be included.   The study designs that will be included in this review includes:   - Experimental   - Randomized Controlled Trials   - Controlled Clinical Trials   - Prospective controlled cohort studies   - Controlled before and after studies - Observational   - Including cross-sectional, longitudinal, case control and cohort studies, wherein participants are:     - Enrolled in OUD treatment     - Can be retrospective or prospective     - Consists of an intervention and control group     - Can be retrospective or prospective     - Consist of intervention and control group | **Study Design**   - Systematic literature reviews - Meta-analyses - Case reports - Case series - Commentary, editorial, opinion piece - Qualitative studies - Economic evaluations |

**iii. eReferences - Included Studies**

1. Ahmadi J. Methadone versus buprenorphine maintenance for the treatment of heroin-dependent outpatients. Journal of substance abuse treatment. 2003;24(3):217-20.
2. Ahmadi J, Ahmadi K, Ohaeri J. Controlled, randomized trial in maintenance treatment of intravenous buprenorphine dependence with naltrexone, methadone or buprenorphine: a novel study. European journal of clinical investigation. 2003;33(9):824-9.
3. Eder H, Fischer G, Gombas W, Jagsch R, Stuhlinger G, Kasper S. Comparison of buprenorphine and methadone maintenance in opiate addicts. European addiction research. 1998;4 Suppl 1:3-7.
4. Gerra G, Borella F, Zaimovic A, Moi G, Bussandri M, Bubici C, et al. Buprenorphine versus methadone for opioid dependence: predictor variables for treatment outcome. Drug and alcohol dependence. 2004;75(1):37-45.
5. Johnson RE, Jaffe JH, Fudala PJ. A controlled trial of buprenorphine treatment for opioid dependence. JAMA. 1992;267(20):2750-5.
6. Kamien JB, Branstetter SA, Amass L. Buprenorphine-naloxone versus methadone maintenance therapy: A randomised double-blind trial with opioid-dependent patients. Heroin Addiction and Related Clinical Problems. 2008;10(4):5-18.
7. Kosten TR, Schottenfeld RS, Ziedonis D, Falconi J. Buprenorphine versus methadone maintenance for opioid dependence. The Journal of Nervous and Mental Disease. 1993;181(6):358-64.
8. Ling W, Wesson DR, Charuvastra C, Klett CJ. A controlled trial comparing buprenorphine and methadone maintenance in opioid dependence. Archives of general psychiatry. 1996;53(5):401-7.
9. Maremmani I, Pani PP, Pacini M, Perugi G. Substance use and quality of life over 12 months among buprenorphine maintenance-treated and methadone maintenance-treated heroin-addicted patients. Journal of substance abuse treatment. 2007;33(1):91-8.
10. Otiashvili D, Piralishvili G, Sikharulidze Z, Kamkamidze G, Poole S, Woody GE. Methadone and buprenorphine-naloxone are effective in reducing illicit buprenorphine and other opioid use, and reducing HIV risk behavior--outcomes of a randomized trial. Drug and alcohol dependence. 2013;133(2):376-82.
11. Pani PP, Maremmani I, Pirastu R, Tagliamonte A, Gessa GL. Buprenorphine: a controlled clinical trial in the treatment of opioid dependence. Drug and alcohol dependence. 2000;60(1):39-50.
12. Proctor SL, Copeland AL, Kopak AM, Herschman PL, Polukhina N. A naturalistic comparison of the effectiveness of methadone and two sublingual formulations of buprenorphine on maintenance treatment outcomes: findings from a retrospective multisite study. Experimental and clinical psychopharmacology. 2014;22(5):424-33.
13. Schottenfeld RS, Pakes JR, Oliveto A, Ziedonis D, Kosten TR. Buprenorphine vs methadone maintenance treatment for concurrent opioid dependence and cocaine abuse. Archives of general psychiatry. 1997;54(8):713-20.

**eReferences - Excluded Studies: Flexible Dose Studies**

1. Fischer G, Gombas W, Eder H, Jagsch R, Peternell A, Stuhlinger G, et al. Buprenorphine versus methadone maintenance for the treatment of opioid dependence. Addiction (Abingdon, England). 1999;94(9):1337-47.
2. Mattick RP, Ali R, White JM, O'Brien S, Wolk S, Danz C. Buprenorphine versus methadone maintenance therapy: a randomized double-blind trial with 405 opioid-dependent patients. Addiction (Abingdon, England). 2003;98(4):441-52.
3. Petitjean S, Stohler R, Deglon JJ, Livoti S, Waldvogel D, Uehlinger C, et al. Double-blind randomized trial of buprenorphine and methadone in opiate dependence. Drug and alcohol dependence. 2001;62(1):97-104.
4. Pinto H, Rumball D, Maskrey V, Holland R. A pilot study for a randomized controlled and patient preference trial of buprenorphine versus methadone maintenance treatment in the management of opiate dependent patients. Journal of Substance Use. 2008;13(2):73-82.
5. Pinto H, Maskrey V, Swift L, Rumball D, Wagle A, Holland R. The SUMMIT trial: a field comparison of buprenorphine versus methadone maintenance treatment. Journal of substance abuse treatment. 2010;39(4):340-52.
6. Soyka M, Zingg C, Koller G, Kuefner H. Retention rate and substance use in methadone and buprenorphine maintenance therapy and predictors of outcome: results from a randomized study. Int J Neuropsychopharmacol. 2008;11(5):641-53.
7. Strain EC, Stitzer ML, Liebson IA, Bigelow GE. Comparison of buprenorphine and methadone in the treatment of opioid dependence. The American journal of psychiatry. 1994;151(7):1025-30.
8. Strain EC, Stitzer ML, Liebson IA, Bigelow GE. Buprenorphine versus methadone in the treatment of opioid-dependent cocaine users. Psychopharmacology (Berl). 1994;116(4):401-6.

**eReferences - Excluded Studies: Outcomes Not Reported**

1. Anglin MD, Conner BT, Annon JJ, Longshore D. Longitudinal effects of LAAM and methadone maintenance on heroin addict behavior. J Behav Health Serv Res. 2009;36(2):267-82.
2. Eder H, Jagsch R, Kraigher D, Primorac A, Ebner N, G. F. Comparative study of the effectiveness of slow-release morphine and methadone for opioid maintenance therapy. Addiction. 2005;100(8):1101–9.
3. Fiellin DA, Schottenfeld RS, Cutter CJ, Moore BA, Barry DT, O’Connor PG. Primary Care–Based buprenorphine taper vs maintenance therapy for prescription opioid dependence: A randomized clinical trial. JAMA Internal Medicine. 2014;174(12):1947-54.
4. Frick U, Rehm J, Zullino D, Fernando M, Wiesbeck G, Ammann J, et al. Long-term follow-up of orally administered diacetylmorphine substitution treatment. European addiction research. 2010;16(3):131-8.
5. Fudala PJ, Bridge TP, Herbert S, Williford WO, Chiang CN, Jones K, et al. Office-based treatment of opiate addiction with a sublingual-tablet formulation of buprenorphine and naloxone. The New England journal of medicine. 2003;349(10):949-58.
6. Hao S-Q, Zhao M, Zhang R-W, Zhang J-C, Zhang J, Feng X-S. The effectiveness comparison of Jitai tablets versus methadone in community-based drug treatment: a 1-year follow-up study. Addictive behaviors. 2013;38(10):2596-600.
7. Kakko J, Svanborg KD, Kreek MJ, Heilig M. 1-year retention and social function after buprenorphine-assisted relapse prevention treatment for heroin dependence in Sweden: a randomised, placebo-controlled trial. Lancet (London, England). 2003;361(9358):662-8.
8. Khodabandeh F, Kahani S, Shadnia S, Abdollahi M. Comparison of the efficacy of methadone maintenance therapy vs. narcotics anonymous in the treatment of opioid addiction: A 2-year survey. International Journal of Pharmacology. 2012;8(5):445-9.
9. Krook AL, Brors O, Dahlberg J, Grouff K, Magnus P, Roysamb E, et al. A placebo-controlled study of high dose buprenorphine in opiate dependents waiting for medication-assisted rehabilitation in Oslo, Norway. Addiction (Abingdon, England). 2002;97(5):533-42.
10. Lee JD, Nunes EV, Novo P, Bachrach K, Bailey GL, Bhatt S, et al. Comparative effectiveness of extended-release naltrexone versus buprenorphine-naloxone for opioid relapse prevention (X:BOT): A multicentre, open-label, randomised controlled trial. The Lancet. 2018;391(10118):309-18.
11. Ling W, Charuvastra C, Kaim SC, Klett CJ. Methadyl acetate and methadone as maintenance treatments for heroin addicts. A veterans administration cooperative study. Archives of general psychiatry. 1976;33(6):709-20.
12. Longshore D, Annon J, Anglin MD, Rawson RA. Levo-alpha-acetylmethadol (LAAM) versus methadone: treatment retention and opiate use. Addiction (Abingdon, England). 2005;100(8):1131-9.
13. Marsch LA, Stephens MAC, Mudric T, Strain EC, Bigelow GE, Johnson RE. Predictors of outcome in LAAM, buprenorphine, and methadone treatment for opioid dependence. Experimental and clinical psychopharmacology. 2005;13(4):293-302.
14. Mokri A, Chawarski MC, Taherinakhost H, Schottenfeld RS. Medical treatments for opioid use disorder in Iran: a randomized, double-blind placebo-controlled comparison of buprenorphine/naloxone and naltrexone maintenance treatment. Addiction (Abingdon, England). 2016;111(5):874-82.
15. Oviedo-Joekes E, Brissette S, Marsh DC, Lauzon P, Guh D, Anis A, et al. Diacetylmorphine versus methadone for the treatment of opioid addiction. The New England journal of medicine. 2009;361(8):777-86.
16. Robertson JR, Raab GM, Bruce M, McKenzie JS, Storkey HR, Salter A. Addressing the efficacy of dihydrocodeine versus methadone as an alternative maintenance treatment for opiate dependence: A randomized controlled trial. Addiction (Abingdon, England). 2006;101(12):1752-9.
17. Sees KL, Delucchi KL, Masson C, Rosen A, Clark HW, Robillard H, et al. Methadone maintenance vs 180-day psychosocially enriched detoxification for treatment of opioid dependence: a randomized controlled trial. JAMA. 2000;283(10):1303-10.
18. Senay EC, Dorus W, Renault PF. Methadyl acetate and methadone. An open comparison. JAMA. 1977;237(2):138-42.
19. Tanum L, Solli KK, Latif Z, Benth JŠ, Opheim A, Sharma-Haase K, et al. Effectiveness of injectable extended-release naltrexone vs daily buprenorphine-naloxone for opioid dependence: A randomized clinical noninferiority trial. JAMA Psychiatry. 2017;74(12):1197-205.
20. Uehlinger C, Déglon J, Livoti S, Petitjean S, Waldvogel D, Ladewig D. Comparison of buprenorphine and methadone in the treatment of opioid dependence. Swiss multicentre study. European addiction research. 1998;4 Suppl 1:13-8.
